# Acute Lower Gastrointestinal Bleeding in an Emergency Department and Performance of the SHA_2_PE Score: A Retrospective Observational Study

**DOI:** 10.3390/jcm10235476

**Published:** 2021-11-23

**Authors:** Titouan Cerruti, Michel Haig Maillard, Olivier Hugli

**Affiliations:** 1 Emergency Department, Lausanne University Hospital, 1011 Lausanne, Switzerland; titouan.cerruti@ghol.ch; 2 Division of Gastroenterology and Hepatology, Lausanne University Hospital, 1011 Lausanne, Switzerland; michel.maillard@chuv.ch; 3 Faculty of Biology and Medicine, Lausanne University, 1011 Lausanne, Switzerland

**Keywords:** lower gastrointestinal bleeding, score, hematochezia

## Abstract

Lower gastrointestinal bleeding (LGIB) is a frequent cause of emergency department (ED) consultation, leading to investigations but rarely to urgent therapeutic interventions. The SHA_2_PE score aims to predict the risk of hospital-based intervention, but has never been externally validated. The aim of our single-center retrospective study was to describe patients consulting our ED for LGIB and to test the validity of the SHA_2_PE score. We included 251 adult patients who consulted in 2017 for hematochezia of <24 h duration; 53% were male, and the median age was 54 years. The most frequent cause of LGIB was unknown (38%), followed by diverticular disease and hemorrhoids (14%); 20% had an intervention. Compared with the no-intervention group, the intervention group was 26.5 years older, had more frequent bleeding in the ED (47% vs. 8%) and more frequent hypotension (8.2% vs. 1.1%), more often received antiplatelet drugs (43% vs. 18%) and anticoagulation therapy (28% vs. 9.5%), more often had a hemoglobin level of <10.5 g/dl (49% vs. 6.2%) on admission, and had greater in-hospital mortality (8.2% vs. 0.5%) (all *p* < 0.05). The interventions included transfusion (65%), endoscopic hemostasis (47%), embolization (8.2%), and surgery (4%). The SHA_2_PE score predicted an intervention with sensitivity of 71% (95% confidence interval: 66–83%), specificity of 81% (74–86%), and positive and negative predictive values of 53% (40–65%) and 90% (84–95%), respectively. SHA_2_PE performance was inferior to that in the original study, with a 1 in 10 chance of erroneously discharging a patient for outpatient intervention. Larger prospective validation studies are needed before the SHA_2_PE score can be recommended to guide LGIB patient management in the ED.

## 1. Introduction

Lower gastrointestinal bleeding (LGIB), defined as bleeding originating distal to the ligament of Treitz, accounts for 30–40% of all gastrointestinal hemorrhages. Its overall annual incidence is 20–87 per 100,000 people, but it is strongly associated with age, increasing by 200 times between the second and ninth decade of life [1,2,3]. LGIB is associated with a mortality rate of 3–15% [1,3,4] and with elderly and polymorbid patients who are at higher risk of complications and death [5,6]. Hematochezia is the most common clinical manifestation of acute LGIB [7] and a frequent cause of emergency department (ED) consultation. Even if the bleeding stops spontaneously, it often leads to investigations during the ED or hospital stay and to significant costs [8]. The challenge for ED physicians when faced with a patient with LGIB is to determine not only the cause and the patient’s prognosis, but also whether admission is necessary for in-hospital investigations or interventions [9]. Decision scores that identify patients suitable for outpatient investigation have been developed for upper gastrointestinal bleeding and have been shown to reduce the rate of hospitalization [10,11]. However, most LGIB scores have focused on identifying patients at risk of major rebleeding [11,12,13], not on identifying those who could be managed as outpatients [11], a gap that the SHA_2_PE score was specifically developed to fill (Appendix A) [9]. This acronym stands for Systolic pressure, Hemoglobin, Anticoagulant or Antiplatelet therapy, Pulse and Emergency room bleeding. A score of ≤1 point indicates a low probability of hospital intervention, allowing for outpatient treatment. However, this score has not yet undergone external validation.

The primary objective of this work was to describe the demographic characteristics, comorbidities, investigations and their timing, treatments, and outcome of LGIB patients who consulted our ED. Our secondary objectives were to assess the predictive performance of the SHA_2_PE score, and assess the respect of one of its components: blood transfusion thresholds.

## 2. Materials and Methods

### 2.1. Study Setting

The ED of the University Hospital of Lausanne has approximately 45,000 annual visits. The hospital serves as a primary care hospital for Lausanne and its boroughs and as a tertiary care hospital for the state and neighboring states in Western Switzerland.

### 2.2. Study Population

Patients ≥ 18 years of age were eligible if they visited the ED between 1 January 2017 and 31 December 2017 with a main complaint at triage of hematochezia occurring within 24 h before their ED arrival. Patients were excluded if they had indicated in the institutional general consent form that they refused the use of their medical data for research purposes or if they had already been included at their index visit.

### 2.3. Data Collection and Outcomes

Medical data were extracted from the various institutional electronic databases, with additions of information from the medical charts, if needed, by manual review by one of the authors (TC). The collected data included demographic and clinical characteristics, medical history, comorbidities, vital signs, laboratory findings, interventions and their timing, use of antiplatelet agents and of anticoagulation, and hospital death. SHA_2_PE in-hospital interventions were defined as in the original article (Appendix B) [9]. All vital signs and laboratory values were the first ones to be registered on ED admission. Missing data were not imputed.

### 2.4. Statistical Analysis

Quantitative variables are presented as means and standard deviation (SD), medians, and interquartile range (IQR); qualitative variables are presented as frequencies and proportions. Comparisons between groups were performed with an unpaired Student-t test, Wilcoxon–Mann–Whitney test, chi-square test, or Fisher’s exact test, as appropriate. A bilateral *p* value of <0.05 was indicative of significant difference. Analyses were performed with STATA, version 15 (StataCorp, College Station, TX, USA).

## 3. Results

### 3.1. Patient Characteristics

**Figure 1:**
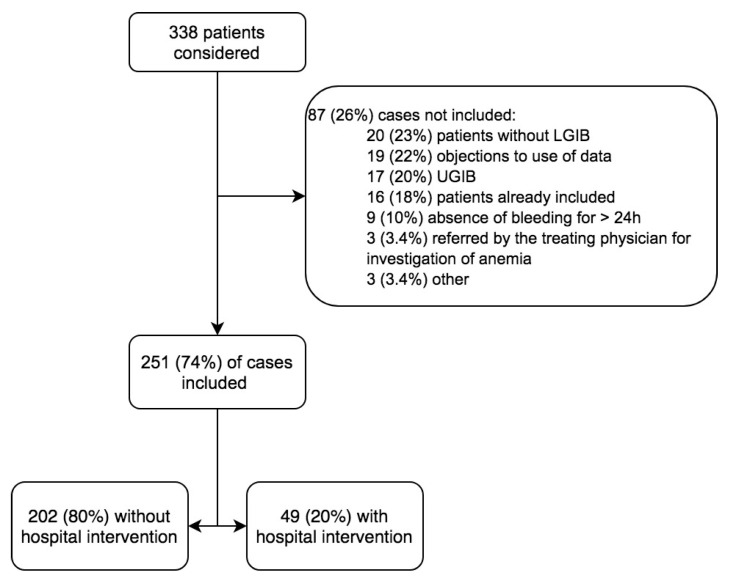
Flowchart. LGIB: lower gastrointestinal bleeding; UGIB: upper gastrointestinal bleeding.

Between 1 January 2017 and 31 December 2017, 338 patients presented to the ED for hematochezia. After exclusions, 251 cases were included (Figure 1). Overall, 49 (20%) received an intervention during their stay (Table 1). They were older than the no-intervention group by an average of 26.5 years, were transported more frequently by ambulance and admitted more often to the intensive care unit, more frequently had bleeding in the ED, had more comorbidities, and were treated more often with antiplatelets or anticoagulants. The death rate was 2% overall, but 16 times higher in the intervention group than in the no-intervention group.

In the intervention group, patients were more often hypotensive on arrival (Table 2); during their stay, they presented with lower blood pressure, were more tachycardic, and more frequently had a shock index of >0.9. On the initial blood work, their hemoglobin (Hb) level was lower and fell markedly during the stay; their international normalized ratio (INR) and creatinine levels were higher.

### 3.2. Investigations and Procedures Not Included in the SHA_2_PE Score 

There were 116 (46%) patients with at least one investigation overall, with a median of one investigation (IQR: 1–2) (Table 3). Endoscopy (esophagogastroduodenoscopy, rectosigmoidoscopy, and colonoscopy) was performed in 100 of 251 patients (40%), with a median of one endoscopy (IQR 0–2) and a maximum of three. In the intervention group, patients were more likely to have had an endoscopy, most commonly a colonoscopy or a rectosigmoidoscopy. Computed tomography angiography (CTA) was performed in one in five patients overall, the rate being twice as high in the intervention group. Outpatient colonoscopy was recommended in 40 patients (16%). The median time to colonoscopy was nearly 32 h, but the time was almost half that in the intervention group (*p* = 0.02). The median time to rectosigmoidoscopy was 16 h, with no significant difference between groups. The median time to CTA was 3–6 times shorter than for lower endoscopy and was similar between groups. The overall length of stay was just short of 12 h, but was 10 times longer for the intervention group than for the no-intervention group. However, 77 of 202 (38%) patients without interventions remained hospitalized for 60 h (IQR 22–112). Administration of blood derivatives and platelets was rare and similar between groups. Tranexamic acid was rarely used and only in the intervention group.

### 3.3. Etiology of LGIB 

The cause of LGIB differed between groups (Table 4). It remained unknown in 38% of patients, with diverticulosis and hemorrhoids being the second most common causes, before anal fissures or iatrogenic complications. The intervention group, which benefited from more investigations, had a lower proportion of unknown LGIB.

### 3.4. Interventions Included in the SHA_2_PE Score 

The most common intervention was blood transfusion (Table 5). A total of 15 of the 34 transfused patients (44%) had a Hb level of ≤9.0 g/dl on admission, including 6 of 15 (40%) with a Hb level of ≤7.0 g/dl. For 21 of 34 patients (62%), their Hb level fell after admission, 31 of 34 (91%) having a Hb level of <9.0 g/dl and 13 of 34 (38%) a Hb level of <7.0 g/dl. According to the defined transfusion limits (Appendix B), 13 of the 34 patients (38%) were transfused at levels above threshold. Of the 34 transfused patients, 12 (35%) had bleeding of diverticular origin but only one had an anorectal pathology. The second most common procedure was endoscopic hemostasis, with clip placements in one third of cases. A maximum of two hemostatic modalities were used in eight of 23 patients (35%). Among the 16 patients who received clip placement, 6 (37%) had bleeding of post-polypectomy origin and 5 (31%) had a diverticular origin. Only two (0.8%) patients required surgical management and survived.

### 3.5. SHA_2_PE Score Performance 

Data needed to calculate the score were available for 209 of 251 patients (83%) (Table 6). Patients with missing data were younger, more frequently self-referred and none were hemodynamically unstable or benefitted from an intervention. Table 6 shows that 14 patients with an intervention were falsely classified as low risk and 29 without intervention as high risk. In our population, sensitivity was 73%, specificity 82%, and area under the receiver operating characteristics (AUROC) curve was 0.77 (95%CI: 0.70–0.84). The negative and positive predictive values were 90% and 56%, respectively. The positive and negative likelihood ratios were 3.95 (95%CI: 2.73–5.72) and 0.34 (95%CI: 0.21–0.53), respectively.

## 4. Discussion

Our retrospective study shows that nearly 50% of patients presenting to the ED with hematochezia benefited from investigations during their hospital admission, and 20% benefited from an intervention. The latter group was older, had more comorbidities, was more often treated with antiplatelet agents or anticoagulants, and was at higher risk of death. Although the vital signs on admission were similar between groups, the interventions group more often developed signs of hemorrhagic shock and a drop in their hemoglobin level. They benefited more often from an endoscopy or CTA, and they had a longer ED length of stay. We found that the performance of the SHA_2_PE score was insufficient to correctly identify patients discharged home without intervention.

Observational studies of patients with acute LGIB have often included only patients admitted to the hospital, and, of those, young patients with benign anorectal pathologies have usually been excluded [3,6,7,14], leading to a selection of more severe cases. In our study, patients were included based on a main concern of hematochezia at triage, before any discharge disposition was made, and explains the younger age of our cohort compared with that in other studies [3,6,7,8]. However, apart from age, the patients’ characteristics were similar to those of previous studies regarding hemodynamic or laboratory parameters [7], the proportion of antiplatelet and anticoagulant treatments at admission [3,5,6,7,15], and comorbidities [3,5,14,16]. Our study confirms the low in-hospital mortality rate of patients with LGIB [3,6,7,17].

The most frequent investigation modality remained lower endoscopy, with more than one third of the patients undergoing it, in agreement with the latest guidelines [18]. In contrast, CAT was performed in 21% of patients, which is two to three times higher than that reported in other studies [3,6,7]. This high percentage reflects the practice of our visceral surgery department [19]. Recently, CTA has taken on a larger role in LGIB investigations, especially for patients with hemodynamic instability or active bleeding [8,18,19,20]. However, its role remains debated in other situations [8,20,21], and further research will be needed to definitively establish its place in LGIB management. Our study also confirms the minor role of angiography, nuclear medicine, and capsule endoscopy in the investigation of LGIB (0.4%, 0.4%, and 0.8%, respectively) [3].

LGIB was of unknown etiology for one third of patients overall, but for nearly one in two of those not investigated during their stay. This high proportion reflects the difficulty of establishing the source of LGIB in the ED, as bleeding has usually stopped at the time of investigations [3,7]. Our proportion of unknown diagnosis may ultimately be lower, as colonoscopy was scheduled as an outpatient procedure for 25 (26%) of the uninvestigated patients. The next most frequent diagnoses were diverticular disease and hemorrhoids, as in other cohorts [3,6,7,14]. We also found that a decreasing proportion of LGIB was due to angiodysplasia [3,15], although the proportion could depend on the rate of endoscopy and increase with outpatient colonoscopies [6].

At 13%, the most frequent intervention was blood transfusion, 38% of which was at an inappropriate threshold based on current guidelines. In England, where gastrointestinal bleeding is the second most common indication for transfusion, studies report up to 80% of inappropriate transfusions despite attempts to implement restrictive thresholds [22]. The recommended threshold is currently 7.0 g/dl in the absence of cardiovascular disease or major bleeding [18,20], based mainly on expert opinion relying on data from trial conducted in UGIB patients [18,20,22]. Because of our retrospective study design, we cannot determine whether these inappropriate transfusions reflect a lack of knowledge by ED physicians or whether they were justified by clinical reasons not documented in the medical chart. However, our data suggest that there may be room for our ED physicians to reduce inappropriate transfusions.

Most scores developed for LGIB aim to identify patients at risk of major bleeding and mortality, with the exception of the recently validated Oakland or the new Birmingham scores, whose main outcome is safe early discharge from the ED of LGIB patients for outpatient management [11,12,17,23,24] (Appendix C). In our population, however, major bleeding and mortality were infrequent, which limits the usefulness of these scores. On the other hand, nearly one in two patients had an investigation, which prolonged their ED length of stay and did not help diagnose a source of LGIB amenable to therapeutic intervention. With chronic overcrowding in most EDs of industrialized nations, the SHA_2_PE would be a welcome addition if it could accurately identify patients unlikely to require inpatient intervention and who could thus be discharged for outpatient investigations. To achieve this goal, the SHA_2_PE score would need a high negative predictive value or a low negative likelihood ratio. In our population, the negative predictive value obtained was 90% with a lower 95% confidence interval (CI) of 84% and was thus worse than in the original SHA_2_PE study [9]. The risk of error could be as high as one in six patients. This lower performance is partly due to the different included populations: only patients who underwent endoscopy were included in the original study [9], whereas we included patients who reported hematochezia at admission. However, our inclusion criteria may be more applicable to clinical decision making in the ED. As older patients were more likely to benefit from an intervention, the addition of age, a criterion of the recent Oakland score [23], could improve score performance. Analysis of the 14 false-negative cases also showed six cases (43%) of post-polypectomy bleeding, a potential additional criterion for the SHA_2_PE if also found by others. The addition of this criteria would increase the negative predictive value to 93% (95%CI: 87–97%), with an AUROC curve of 0.80 (95%CI: 0.65–0.82). Furthermore, in our population, 12 (38%) patients were transfused inappropriately, thus lowering the performance of the SHA_2_PE score. On the other hand, 32 (15%) patients did not received a transfusion, despite it being recommended by guidelines. If all patients had been transfused according to the recommended indications, the negative predictive value would have been 87% (95%CI: 80–92%), with an AUROC curve of 0.70 (95%CI: 0.63–0.78).

If patients with a missing SHA_2_PE score are considered as having a low probability of intervention, a reasonable assumption given their ED clinical condition, the negative predictive value would increase to 92% (95%CI: 88–96%), with an AUROC curve of 0.78 (95%CI: 0.71–0.85). These values are high, but still too low to recommend its use in the ED, compared to the validated Oakland score that has a negative predictive value of 99% for score ≤8 points (calculated from [23] (see also Appendix C for a comparison of the AUROC curves from different scores). As an example of misclassification, a 70-year-old patient was classified as low-risk, after presenting with hematochezia and syncope; he benefited from a colonoscopy the following day that revealed extensive diverticulosis but failed to show the source of bleeding. He bled profusely again later the same day. On CTA, a large caecal bleed was diagnosed requiring a hemicolectomy. The patient survived.

Our study has some limitations. First, we used a motive at triage as an inclusion criterion, and some LGIB could have been initially triaged with another criterion. In 2017, 27 patients with a final diagnosis of LGIB were triaged with different motives, or 8% of our cohort (338 patients). Thus, our inclusion criterion allowed us to include the vast majority of LGIB. Second, because of our retrospective design, the variables required for the calculation of the SHA_2_PE score were missing for 17% of patients. These patients were unlikely to require a hospital-based intervention. Third, we included patients who could have had hematochezia in the form of blood on toilet paper, which was an exclusion criterion in the original study [9]. Consequently, the specificity, negative predictive value, and negative likelihood ratio could have been higher if these patients had been excluded. Finally, it is recommended that 100–200 events are required to externally validate a prognostic model with sufficient precision [25]. Our study only had 49 events, resulting in relatively wide 95%CI.

## 5. Conclusions

Our study shows that one in two patients admitted for hematochezia benefited from investigations, that one in five benefited from an intervention and that their in-hospital mortality was very low. Elderly patients with pre-existing co-morbidities benefited from interventions more often, leading to significantly longer lengths of stay. In our population, missing data and non-compliance with recommended transfusion thresholds interfered with the determination of the score performance. With these limitations in mind, we found that the negative predictive value of the SHA_2_PE score was too low to safely identify patients who were suitable for outpatient investigations and interventions. Our data suggest that the performance of the score could be improved by the addition of two factors: age and post-procedural bleeding. An implementation study with the original score, or the score with our suggestions for improvement, is needed to provide a definitive proof regarding the score performance.

## Figures and Tables

**Table 1:** Patient characteristics.

	All	Without Intervention	Intervention	*p*
	*n* = 251	*n* = 200	*n* = 51	
Male, *n* (%)	134 (53)	102 (51)	32 (63)	0.16
Median age, years (IQR)	54 (37–76)	48 (34–74)	75 (62–82)	<0.001
Admission mode, *n* (%)				
Pedestrian	193 (77)	167 (84)	26 (51)	<0.001
Ambulance	47 (19)	25 (13)	22 (43)	
Unknown	11 (4.4)	8 (4.0)	3 (5.9)	
Resuscitation room admission, *n* (%)	4 (1.6)	0	4 (7.8)	0.002
Hemorrhage in the ED, *n* (%)	39 (16)	16 (8.1)	23 (45)	<0.001
Comorbidities, *n* (%)				
Hypertension	90 (36)	57 (29)	33 (65)	<0.001
Diabetes	30 (12)	16 (8.0)	14 (27)	<0.001
Coronary heart disease	28 (11)	14 (7.0)	14 (27)	<0.001
Heart failure	7 (2.8)	3 (1.5)	4 (7.8)	0.033
Atrial fibrillation	24 (9.6)	13 (6.5)	11 (22)	0.03
Acute vascular accident				0.009
Stroke	12 (4.8)	7 (3.5)	5 (9.8)	
Myocardial infarction	8 (3.2)	4 (2.0)	4 (7.8)	
Stroke + Myocardial infarction	4 (1.6)	2 (1.0)	2 (3.9)	
Chronic obstructive pulmonary disease	8 (3.1)	5 (2.5)	3 (5.8)	0.21
Active smoking	23 (9.2)	19 (9.5)	4 (7.8)	0.99
Dementia	6 (2.4)	5 (2.5)	1 (2.0)	0.99
Acute renal failure	25 (10)	13 (6.5)	12 (24)	0.001
Inflammatory bowel disease	10 (4)	7 (3.5)	3 (5.9)	0.43
Diverticulosis	25 (10)	17 (8.5)	8 (16)	0.19
Cirrhosis	8 (3.2)	6 (3.0)	2 (3.9)	0.67
Cancer				0.08
Localized, digestive, *n* (%)	3 (1.2)	1 (0.5)	2 (3.9)	
Nondigestive localized, *n* (%)	7 (2.8)	6 (3.0)	1 (2.0)	
Metastatic, *n* (%)	4 (1.6)	2 (1)	2 (3.9)	
History of LGIB, *n* (%)	42 (17)	28 (14)	14 (27)	0.034
Treatment at entry, *n* (%)				
Antiplatelet	57 (23)	34 (17)	23 (45)	<0.001
Anticoagulant	33 (13)	18 (9)	15 (29)	<0.001
Anti-vitamin K	14 (5.6)	6 (3.0)	8 (16)	
DOAC	14 (5.6)	10 (5.0)	4 (7.8)	
LMWH	5 (2)	2 (1)	3 (5.8)	
NSAIDs, *n* (%)	25 (10)	18 (9.0)	7 (14)	0.30
Deaths, *n* (%)	5 (2.0)	1 (0.5)	4 (7.8)	0.007

IQR: interquartile range; ED: emergency department; DOAC: direct oral anticoagulant; LMWH: low molecular weight heparin; NSAIDs: nonsteroidal anti-inflammatory drugs.

**Table 2:** Vital signs and biological workup at admission.

	All *n* = 251	Without Intervention *n* = 200	Intervention *n* = 51	*p*
Vital signs on admission				
SBP (*n* = 233), mmHg (SD)	132 (20)	132 (19)	133 (23)	0.89
SBP < 100 mmHg, *n* (%)	6 (2.6)	2 (1.1)	4 (7.8)	0.022
DBP (*n* = 233), mmHg (SD)	75 (13)	76 (12)	69 (15)	<0.001
HR (*n* = 231),/min (SD)	79 (15)	78 (14)	82 (19)	0.06
HR > 100/min, *n* (%)	16 (6.9)	12 (6.6)	4 (7.8)	0.76
Shock index > 0.9 (*n* = 231), *n* (%)	6 (2.6)	3 (1.7)	3 (5.9)	0.12
Respiratory rate at entry (*n* = 85),/min (SD)	17.1 (2.7)	17.0 (2.4)	17.2 (3.2)	0.76
SatO_2_ (*n* = 218)_,_ % (SD)	98 (2)	98 (2)	98 (2)	0.92
Extreme vital signs during the stay				
Lowest SBP (*n* = 136), mmHg (SD)	121 (19)	123 (18)	111 (20)	0.005
SBP < 100 mmHg, *n* (%)	14 (10)	8 (7.1)	6 (25)	0.018
Highest HR (*n* = 231),/min (SD)	85 (19)	83 (17)	95 (21)	<0.001
HR > 100/min, *n* (%)	36 (16)	22 (12)	14 (28)	0.01
Shock index > 0.9 (*n* = 135), *n* (%)	20 (15)	10 (9.0)	10 (42)	<0.001
Biology at admission				
Hemoglobin (*n* = 226), g/dl (SD)	12.9 (2.4)	13.6 (1.8)	10.5 (2.9)	<0.001
<10.5, *n* (%)	35 (15)	9 (5.1)	26 (51)	<0.001
10.5–12.0, *n* (%)	30 (13)	22 (13)	8 (16)	
>12.0, *n* (%)	161 (71)	144 (82)	17 (33)	
Platelets (*n* = 226), G/L (SD)	250 (83)	248 (79)	255 (95)	0.58
INR (SD) (*n* = 194), IU (SD)	1.2 (0.5)	1.1 (0.3)	1.3 (0.9)	0.003
PTT (*n* = 193), seconds (SD)	30.4 (6.3)	30.1 (6.0)	31.3 (7.1)	0.23
Creatinine (*n* = 223), μmol/L (IQR)	80 (66–100)	78 (66–98)	96 (72–121)	0.005
Urea (*n* = 83), mmol/l (IQR)	5.8 (4.4–8.3)	5.4 (4.1–7.1)	6.6 (4.7–11.9)	0.06
Biology: extreme values during stay				
Lowest Hb (*n* = 224), g/dl (SD)	11.9 (3.0)	12.9 (2.3)	8.5 (2.4)	<0.001
<10.5, *n* (%)	71 (32)	29 (17)	42 (82)	<0.001
10.5–12.0, *n* (%)	32 (14)	29 (17)	3 (5.9)	
>12.0, *n* (%)	121 (54)	115 (66)	6 (12)	

SD: standard deviation; SBP: systolic blood pressure; DBP: diastolic blood pressure; HR: heart rate; SatO_2_: oxygen saturation; INR: international normalized ratio; PTT: partial thromboplastin time; IQR: interquartile range; Hb: hemoglobin.

**Table 3:** Investigations and interventions not included in the SHA_2_PE score.

	All *n* = 251	Without Intervention *n* = 200	Intervention *n* = 51	*p*
Investigation, *n* (%)	116 (46)	70 (35)	46 (94)	<0.001
Colonoscopy	66 (26)	33 (17)	33 (65)	<0.001
Rectosigmoidoscopy	55 (22)	27 (14)	28 (55)	<0.001
CTA	54 (21)	33 (17)	21 (41)	0.001
Esogastroduodenoscopy	22 (8.8)	10 (5.0)	12 (24)	<0.001
Angiography	1 (0.4)	0	1 (2.0)	0.20
Nuclear medicine	1 (0.4)	0	1 (2.0)	0.20
Capsule endoscopy	2 (0.8)	1 (0.5)	1 (2.0)	0.37
Time to investigation, h (IQR)				
Colonoscopy	31.9 (16.9–52.1)	37.9 (25.4–73.0)	18.2 (12.0–46.1)	0.01
Rectosigmoidoscopy	16.1 (7.4–25.2)	18.7 (7.5–39.8)	14.6 (5.4–21.5)	0.15
CTA	5.3 (2.7–30.7)	5.0 (2.9–21.3)	5.3 (2.4–36.7)	0.84
Esogastroduodenoscopy	12.1 (5.5–20.3)	17.9 (7.8–21.5)	5.5 (4.3–17.3)	0.06
Length of stay, h (IQR)	11.3 (4.0–61.4)	6.3 (3.6–25.2)	65.8 (35.0–105.8)	<0.001
Treatment, *n* (%)				
Platelet transfusion	1 (0.4)	0	1 (2.0)	0.20
Fresh frozen plasma	8 (3.2)	1 (0.5)	7 (14)	<0.001
Prothrombin concentrate complex	1 (0.4)	0	1 (2.0)	0.20
Fibrinogen	1 (0.4)	0	1 (2.0)	0.20
Tranexamic acid	4 (1.6)	0	4 (7.8)	0.002

CTA: computed tomography angiography; IQR: interquartile range.

**Table 4:** Etiology of LGIB.

	All *n* = 251	Without Intervention *n* = 200	Intervention *n* = 51	*p*
**Diagnostics, *n* (%)**				
Unknown	96 (38)	88 (44)	8 (16)	<0.001
Diverticulosis	35 (14)	20 (10)	15 (29)	0.001
Hemorrhoids	35 (14)	33 (17)	2 (3.9)	0.023
Anal fissure	21 (8)	21 (11)	0	0.018
Post-polypectomy/iatrogenic	18 (7.2)	7 (3.5)	11 (22)	<0.001
Infectious colitis	15 (6)	14 (7.0)	1 (2.0)	0.32
Inflammatory bowel disease	10 (4.0)	7 (3.5)	3 (5.9)	0.43
Ischemic/post-radiation colitis	9 (3.6)	5 (2.5)	4 (7.8)	0. 09
Angiodysplasia	4 (1.6)	0	4 (7.82)	0.002
Polyp	4 (1.6)	3 (1.5)	1 (2.0)	0.99
Cancer	2 (0.7)	1 (0.5)	1 (2.0)	0.37
Trauma	2 (0.7)	1 (0.5)	1 (2.0)	0.37

**Table 5:** Interventions included in the SHA_2_PE score.

	All *n* = 51	SHA_2_PE > 1 Point *n* = 37	SHA_2_PE ≤ 1 Point *n* = 14	*p*
Type of intervention, *n* (%)				
Blood transfusion	34 (67)	29 (78)	5 (36)	0.007
Inappropriate transfusion	13 (24)	10 (75)	3 (25)	0.345
Endoscopic treatments, *n* (%)	23 (45)	14 (38)	9 (64)	0.005
Clip	16 (31)	10 (27)	6 (43)	
Adrenaline	8 (16)	4 (11)	4 (29)	
Thermocoagulation	6 (12)	3 (8.1)	3 (21)	
Banding	1 (2.0)	1 (2.9)	0	
Interventional radiology, *n* (%)	4 (8.2)			
Surgery, *n* (%)				0.49
Hemicolectomy	1 (2.0)	0	1 (7.1)	
Hemostasis	1 (2.0)	1 (2.9)	0	

**Table 6:** SHA_2_PE score performance.

	SHA_2_PE > 1 Point (High Probability)	SHA_2_PE ≤ 1 Point (Low Probability)	Predictive Value (%)
Intervention, *n*	37	14	Positive: 56 (95% CI: 43–68)
No intervention, *n*	29	129	Negative: 90 (95% CI: 84–95)
	Sensitivity (%) 73 (95% CI: 58–84)	Specificity (%) 82 (95% CI: 75–87)	

CI: confidence interval.

## Data Availability

The data are not publicly available, as participants of this study did not agree for their data to be shared publicly.

## Appendix A

**Table A1:** SHA_2_PE score.

Item	Points
Systolic pressure < 100 mmHg	1
Hemoglobin value	
<105 gr/L	2
105–120 gr/L	1
Antiplatelet therapy	1
Anticoagulant therapy	1
Pulse > 100/min	1
Emergency room bleeding	1

A score of ≤1 point indicates very low probability of requiring hospital intervention.

## Appendix B

**Table A2:** Study definitions.

Study Definitions
**Hemorrhage in the Emergency Department** Objective bleeding in the emergency department within the first 4 h of presentation. This definition does not include blood on the glove after digital rectal examination.
**Shock**
Heart rate (HR) of >100/min associated with systolic blood pressure (SBP) of <100 mmHg
**Major hemorrhage**
Bleeding leading to SBP of <90 mmHg or HR of >110/min
**Appropriate threshold for blood transfusion**
Hemoglobin (Hb) < 7.0 g/dl in patients without major hemorrhage (see above) or major comorbidity (mainly cardiovascular). For these two situations, a threshold of Hb < 9.0 g/dl was considered adequate.
**In-hospital mortality**
Any cause of death during the patient’s stay
**Intervention**
Blood transfusion, endoscopic hemostasis, embolization by interventional radiology or surgery

## Appendix C

**Table A3:** C-statistics for the SHA_2_PE compared to previously published models for safe discharge (adapted from ref [11]).

Score	Original Predicted Outcome	C-Statistics for Safe Discharge (95%CI)	References
Oakland (Derivation)	Safe discharge	0.84 (0.82–0.86)	Oakland K et al. Lancet Gastroenterol Hepatol. 2017;2:635–643.
Glasgow-Blatchford	Need for intervention	0.80 (0.78–0.82)	
AIMS65	Length of stay and mortality	0.62 (0.60–0.64)	
BLEED	In-hospital complications and mortality	0.63 (0.61–0.65)	
STRATE	Severe hemorrhage	0.69 (0.66–0.71)	
NOBLADS	Severe hemorrhage, transfusion, length of stay, need for intervention	0.65 (0.63–0.67)	
Pre-endoscopy Rockall	Death and rebleeding	0.64 (0.61–0.66)	
Oakland (Validation)	Safe discharge	0.87 (0.87–0.87)	Oakland K, et al. JAMA Netw Open. 2020;3:e209630
Birmingham (Derivation)	Safe early discharge	0.86 (0.82–0.90)	Smith SCL et al. Int J Colorectal Dis. 2020;35:285–293
Birmingham (Validation)	Safe early discharge	0.29 (0.24–0.34)	This publication
SHA_2_PE (Derivation)	Need for intervention	0.83 (NA)	Hreinsson JP et al. Scand J Gastroenterol. 2018;53:1484–1489
SHA_2_PE (Validation)	Need for intervention	0.77 (0.70–0.84)	This publication
